# Medical News and Items

**Published:** 1876-05

**Authors:** 


### Medical News and Items.

ILLINOIS STATE MEDICAL SOCIETY. — The Twenty-sixth Annual Meeting of this Society will be held in Urbana, Champaign County, May 16th, at 10 o'clock A. M.

---

AMERICAN MEDICAL ASSOCIATION.—The 27th Annual Session will be held in the city of Philadelphia, Pa., on Tuesday, June 6, 1876, at 11 A.M.

"The delegates shall receive their appointment from permanently organized State Medical Societies, and such County and District Medical Societies as are recognized by *representation in their respective State Societies*, and from the Medical Department of the Army and Navy of the United States."

"Each State, County, and District Medical Society entitled to representation shall have the privilege of sending to the Association one delegate for every ten of its regular resident members, and one for every additional fraction of more than half that number ; *Provided*, however, that the number of delegates for any particular State, territory, county, city or town shall not exceed the ratio of one in ten of the resident physicians who may have signed the Code of Ethics of the Association."

☞ Secretaries of Medical Societies as above designated are earnestly requested to forward, *at once*, lists of their delegates, in order that the Committee of Arrangements may be enabled to form some idea of the number likely to be present.

SECTIONS.

"The chairmen of the several sections shall prepare and read in the general sessions of the Association, papers on the advances and discoveries of the past year in the branches of science included in their respective sections. * * * "—BY-LAWS, Art. II., Sect. 4.

Practice of Medicine, Materia Medica, and Physiology : Dr. Francis G. Smith, Philadelphia, Pa., Chairman ; Dr. B. A. Vaughan, Columbus, Miss., Secretary. Committee appointed to report to this Section—On Clinical Observations : Dr. N. S. Davis, Ill., Chairman ; Dr. H. A. Johnson, Ill.; Dr. J. B. Johnson, Mo.

Obstetrics and Diseases of Women and Children: Dr. S.C. Busey, Washington, D. C., Chairman ; Dr. Robert Battey, Atlanta, Ga., Secretary. Committee appointed to report to this Section—On the Connection of the Hepatic Circulation with Uterine Hyperæmias, Fluxions, and Inflammations: Dr. L. F. Warner, Mass.

Surgery and Anatomy: Dr. Alonzo Garcelon, Lewiston, Me., Chairman ; Dr. E. T. Easley, Dallas, Texas, Secretary.

Medical Jurisprudence, Chemistry, and Psychology : Dr. E. Lloyd Howard, Baltimore, Md., Chairman ; Dr. V. L. Hurlbut, Chicago, Ill., Secretary.

State Medicine and Public Hygiene : Dr. R. C. Kedzie, Lansing, Mich. Chairman ; Dr. Ezra M. Hunt, Metuchen, N. J., Secretary. Committee to report to this Section—On Form of Bill to Establish a National Department of Public Health at Washington : Dr. H. B. Baker, Mich., Chairman ; Dr. H. A. Johnson, Ill. ; Dr. J. M. Toner, D. C.

☞ "Papers appropriate to the several sections, in order to secure consideration and action, must be sent to the Secretary of the appropriate section at least one month before the meeting which is to act upon them. It shall be the duty, of the Secretary to whom such papers are sent, to examine them with care, and, with the advice of the Chairman of his section, to determine the time and order of their presentation, and give due notice of the same. * * * "—BY-LAWS, Art. II, Sect. 5.

The following Committees are expected to report: On Mechanism of Accommodation of the Eye: Dr. D. S. Reynolds, Ky., Chairman.

On New Remedies: Dr. Austin Flint, Jr., N. Y., Chairman.

On the Medical and Surgical Uses of the Aspirator: Dr. E. S. Gaillard, Ky., Chairman.

On Influence of Climate on Pulmonary Diseases in Minnesota: Dr. Franklin Staples, Minn., Chairman.

On the same in Colorado: Dr. Chas. Denison, Col., Chairman.

On the same in Florida: Dr. E. T. Sabal, Fla., Chairman.

On Proper Legislation to Prevent the Spread of Syphilis: Dr. Samuel D. Gross, Pa., Chairman.

On Prize Essays: Dr. Samuel D. Gross, Pa., Chairman.

On Necrology: Dr. S. C. Chew, Md., Chairman.

On Rank of Medical Corps of the Army: Dr. H. A. Johnson, Ill., Chairman.

W. B. ATKINSON, *Permanent Sec'y.*

PHILADELPHIA, 1400 Pine St., S. W. çor. Broad.

---

CHICAGO MEDICAL PRESS ASSOCIATION.—The Directors wish to express thanks for the following books received. From Dr. Philip Adolphus, Chicago, 44 vols., as follows:

Cooper's Lectures.
Howshep on the Rectum.
Bell on Ulcers.
Cooper's Surgery, 2 vols.
Strohmeyer & Eshmarch on Gun Injuries.
Guthrie, Military Surgery.
Carmichael on Venereal.
Cazenave on the Skin.
Bateman, Synopsis of Veneral Diseases.
Travers on Irritation.
Rogerson on Inflammation.
Andral on Chest Diseases.
Andral, Diseases of Abdomen.
Abercrombie on the Brain.
Abercrombie on the Stomach.
Budd on the Liver.
Armstrong on Typhus Fever.
Ayre on Dropsy.
Armstrong on Fevers.
Andral on the Encephalon.
Bichat on Pathological Anatomy.
Andral on the Blood in Disease.
Philip on Acute and Chronic Diseases.
Beclard's General Anatomy.
Bell's Anatomy, 3 vols.
Parson's Anatomical Preparations.
Brechard's Physiological Researches.
Broussais' Physiology.
Wilson on Vital Functions.
Blumenbach's Physiology.
Richenad's Physiology.
Beain's Therapeutics.
Paris' Pharmacologia.
Ellis' Medical Formulary.
Paris on Diet.
Combe on Infancy.
Althaus' Medical Electricity.
Brand and Taylor's Chemistry.
Brodie on Joints.
Prout on Calculus.
Report of Board of Health of Chicago for 1867–9.

Also the following books and pamphlets from Dr. Hamill:

Principal Diseases of the Valley of North America. 1849. D. Drake, M.D. 1 vol.
Second Series Principal Diseases of the Valley of North America. 1850. D. Drake, M.D. 1 vol.
American Journal Medical Sciences for 1844–5. 4 vols. in 2.
Principles of Pathology and Practical Physic. By John Mackintosh. Edition 1837. 2 vols.
Bell and Stokes. 10 vols. Gooch on Females. 1832. 1 vol.

Art of Preventing Disease and Restoring Health. By Geo. Wallis, M.D., S.M.S. 1794. 1 vol.
Western Journal Medical Science for 1834–5. Transactions American Medical Association for those years. 1 vol.
Combe's System of Phrenology. 1 vol.
Medical and Surgical History of the War of the Rebellion: Surgeon Barnes' Report. 1870. 2 vols.
Cholera Epidemic of 1873 in the United States. J. M. Woodworth, M.D. 1 vol.
Circular of War Department by Surgeon Barnes. 1870. 1 vol.
Statistics—Population. 1870. 1 vol.
Mortuary Statistics. 1860. 1 vol.
Population Statistics. 1860. 1 vol.
Vital Statistics. 1870. 1 vol.
Population Statistics. 1870. 1 vol.
Wister's Anatomy. 1835. 2 vols.
Library of Practical Medicine. 1832. 1 vol.
Thompson's Conspectus. 1846. 1 vol.

PAMPHLETS.

Chicago Medical Journal for 1868–9. 2 vols.
Medical Examiner for 1868–9–70–71–72–73–74–75.
Transactions of Illinois State Med. Society for 1860–64–67–68–69–70–71–74.
And sundry Medical Journals.

